# Pleural Effusion Phenotype and Ultrasound Guidance Approach Associated with Unplanned Pigtail Pleural Catheter Removal in an Adult Intensive Care Unit: A Single-Center Retrospective Cohort Study

**DOI:** 10.3390/life16081331

**Published:** 2026-08-14

**Authors:** Wei-Hung Chang, Kuan-Pen Yu, Li-Kuo Kuo, Chung Lee

**Affiliations:** 1 Department of Critical Care Medicine, MacKay Memorial Hospital, Taipei 10449, Taiwan; peacejaycool@gmail.com (W.-H.C.);; 2 Department of Medicine, Mackay Medical College, New Taipei City 25245, Taiwan; 3 Department of Chest Medicine, Taipei Tzu Chi Hospital, Buddhist Tzu Chi Medical Foundation, Taipei 10449, Taiwan

**Keywords:** pleural effusion, pigtail catheter, thoracic ultrasound, intensive care unit, empyema, malignant pleural effusion, hemothorax

## Abstract

Background: Pleural pigtail catheters are widely used in intensive care units (ICUs), but factors associated with unplanned removal remain incompletely characterized. Methods: We conducted a single-center retrospective cohort study of adult ICU catheter-placement episodes from December 2020 to March 2025. Effusions were grouped as complex (exudative, empyema, malignant, or malignancy-related) or simple (transudative or hemothorax). The primary outcome was unplanned removal for a catheter-related complication or escalation to surgery. Associations were evaluated using Firth penalized logistic regression. Results: Among 175 screened episodes, 140 were analyzed; unplanned removal occurred in 12 (8.6%). Complex effusions (adjusted odds ratio [aOR] 6.50, 95% confidence interval [CI] 1.59–26.55) and ultrasound-assisted insertion (aOR 10.72, 95% CI 1.92–59.94) were associated with higher odds of unplanned removal. Reclassifying hemothorax as complex attenuated but did not reverse the phenotype association (aOR 4.84, 95% CI 1.17–19.93). The ultrasound association may reflect case selection, operator workflow, or procedural complexity rather than causality. Because 33 death-related episodes were excluded, the 8.6% rate applies to episodes with classifiable non-death endpoints, not all screened episodes. Conclusions: These findings are hypothesis-generating and insufficient to change clinical practice without prospective multicenter validation.

## 1. Introduction

Pleural effusion is a frequent bedside problem in adult intensive care units (ICUs), where it often coexists with respiratory failure, sepsis, postoperative care, malignancy, renal replacement therapy, and major fluid shifts. In the ICU, a pleural collection is rarely an isolated radiographic abnormality. It can affect oxygenation, ventilator mechanics, diagnostic decision-making, and the timing of escalation or de-escalation of organ support. Earlier ICU studies showed that pleural effusions are common and clinically meaningful, but also that drainage decisions are highly contextual and heterogeneous [1,2]. A systematic review in mechanically ventilated patients suggested that pleural drainage may improve oxygenation, yet the evidence base remained dominated by observational studies and clinically diverse patient groups [3]. Thus, the practical question at the bedside is not simply whether pleural fluid exists, but whether a specific pleural process, in a specific ICU context, will follow an uncomplicated catheter-drainage course.

For ICU teams, the catheter course is a practical endpoint. Unplanned removal may expose patients to repeat imaging, repeat pleural puncture, persistent respiratory compromise, delayed source control, or transfer to procedural services at a time when physiologic reserve is limited. It also affects workflow: nurses and physicians must troubleshoot drainage systems, verify catheter position, reassess pleural residuals, and communicate whether the original procedural plan remains viable. These downstream consequences are not captured well by studies that focus only on immediate pneumothorax or bleeding after access. A catheter can be inserted safely and still fail as a drainage strategy if the pleural process is organized, the catheter migrates, or the clinical course requires escalation. For this reason, episode-level outcomes may be more informative for ICU operations than insertion-day complication rates alone.

Pleural effusions are etiologically and biologically heterogeneous. Light’s criteria remain the canonical framework for separating transudates from exudates, but the post-insertion behavior of a pleural catheter may depend on additional features that are not fully captured by a binary transudate–exudate distinction [4]. Pleural infection, for example, may be loculated, septated, viscous, and inflammatory; catheter drainage may be incomplete despite apparently successful access; and escalation to fibrinolytic therapy, additional drainage, or surgery may become necessary [5,6]. Malignant pleural effusion is also a high-burden entity in which recurrence, symptom palliation, trapped lung physiology, and procedural burden influence management choices [7]. Hemothorax introduces a different set of concerns related to clot burden and the timing of intervention. These differences support the concept that catheter-course outcomes should be analyzed not only by insertion technique, but also by the clinical phenotype of the fluid collection being drained.

Thoracic ultrasound is central to contemporary pleural practice. It can confirm the presence of fluid, estimate the size and distribution of the collection, identify septations or complex echogenic material, and help select a safer access site. Procedural complications such as pneumothorax, bleeding, and visceral injury are clinically consequential in critically ill patients, and guidance documents have increasingly emphasized image-based assessment, procedural planning, and operator competency [8,9,10,11,12]. Observational evidence also suggests that ultrasound guidance can reduce complications and costs in pleural procedures such as thoracentesis [13]. However, the label ‘ultrasound-guided’ may obscure important operational differences. A procedure performed after pre-procedure ultrasound localization is not the same workflow as pleural access and catheter advancement under continuous real-time sonographic visualization.

Small-bore wire-guided pleural catheters, including pigtail catheters, are attractive in the ICU because they can be inserted through a minimally invasive Seldinger technique and are often used by intensivists or chest physicians at the bedside. Nevertheless, the success of a pleural catheter is not determined at skin entry alone. Displacement, malposition, poor drainage, blockage, persistent loculation, and the need for surgical escalation can occur later in the drainage episode [14,15,16]. Most of the procedural safety literature emphasizes immediate insertion-related complications, whereas the broader catheter course—from placement to removal—has been less consistently studied. This distinction matters operationally: the clinician must decide how closely to reassess the catheter, when to obtain repeat imaging, and when the initial catheter strategy should be abandoned.

This gap is particularly relevant for retrospective ICU datasets because the unit of documentation is often the procedure rather than the disease trajectory. A procedure database can accurately record the catheter, laterality, ultrasound approach, and removal reason, but it may not contain every nuance of pleural fluid biochemistry, radiographic evolution, or clinician rationale. A pragmatic analytic approach must therefore respect the structure of the data while still asking a question that matters clinically. Grouping effusions by procedural phenotype grouping is one way to balance biological detail with reproducible bedside classification. It does not replace disease-specific analysis, but it creates a workable first layer of risk stratification for sparse real-world data.

In this single-center retrospective cohort study, we evaluated adult ICU pigtail pleural catheter placement episodes and asked whether a pragmatic pleural effusion phenotype grouping and ultrasound guidance approach were associated with episode-level unplanned catheter removal. We defined unplanned removal as removal for a documented catheter-related complication or escalation to surgery, intentionally capturing non-elective deviation from the planned catheter-drainage pathway rather than only immediate mechanical complications. We hypothesized that complex pleural effusion grouping and ultrasound-assisted insertion, compared with real-time ultrasound-guided insertion, would be associated with a higher likelihood of unplanned catheter removal.

By focusing on a procedure-level cohort, this study was designed to generate immediately testable hypotheses for ICU pleural-drainage governance. The intended output is not a bedside scoring tool, but a structured signal that can inform prospective data collection, internal audit, local quality-improvement cycles, and future multicenter validation across different procedural environments. This framing also helps reviewers distinguish the clinical message from overclaiming based on a sparse retrospective signal.

## 2. Materials and Methods

### 2.1. Study Design, Setting, and Reporting

This was a single-center retrospective cohort study conducted in the adult ICUs of Taipei MacKay Memorial Hospital, Taipei, Taiwan. The study used procedure-level data from an institutional ICU pleural procedure database linked with electronic health record (EHR) documentation. The manuscript was prepared in accordance with the Strengthening the Reporting of Observational Studies in Epidemiology (STROBE) reporting approach, with emphasis on transparent cohort assembly, exposure definition, outcome definition, missing data description, and sensitivity analysis.

The clinical setting was a real-world adult critical care environment in which pigtail pleural catheters were placed for pleural effusion or hemothorax according to local clinical practice. Because the study was retrospective and observational, no procedural assignment, device selection, or post-placement management strategy was imposed by the study protocol. The analysis therefore estimates associations between recorded clinical-procedural exposures and catheter-course outcomes rather than randomized causal effects.

### 2.2. Study Population and Cohort Assembly

Consecutive pigtail pleural catheter placement episodes performed for drainage of pleural effusion or hemothorax between 28 December 2020 and 18 March 2025 were screened. Follow-up for each episode extended from catheter placement to catheter removal; the latest recorded catheter removal date in the source data was 27 March 2025. The index date was defined as the catheter placement date for each episode.

The analytic unit was the catheter placement episode rather than the individual patient. This choice was prespecified because the exposure classification, ultrasound guidance approach, catheter dwell time, and catheter removal reason were recorded at the procedure level. A single patient could therefore contribute more than one eligible episode if more than one catheter placement occurred during the study period. To evaluate the robustness of the primary analysis to repeated procedures, a sensitivity analysis was restricted to the first eligible episode per patient, defined as the earliest eligible placement date.

The episode-level framework was chosen to preserve the temporal relationship between catheter-specific exposures and catheter-specific outcomes. If a patient had bilateral procedures, sequential procedures, or recurrent pleural drainage, each catheter episode could have a different laterality, insertion approach, dwell time, and removal reason. Collapsing these observations to the patient level would have discarded clinically relevant procedural information and introduced ambiguity about which exposure corresponded to which outcome. The trade-off is that the primary analysis may include correlated observations from the same patient, which is why the first-episode sensitivity analysis is important for interpretation.

Episodes were eligible if the patient was at least 18 years of age and underwent ICU pigtail pleural catheter placement for pleural effusion or hemothorax. For the primary analysis, episodes were excluded if the recorded catheter removal reason was death or if pleural effusion classification was unknown. This exclusion was made to create an analytic cohort in which the primary catheter-course endpoint could be classified according to the prespecified outcome definition. It should not be interpreted as equating death with planned catheter completion, and the limitation related to death as a competing event is addressed in the Discussion.

### 2.3. Data Sources and Variables

Data were obtained from structured fields in the ICU pleural procedure database and linked EHR documentation. Baseline variables collected at or before catheter placement included age, sex, ICU unit, effusion laterality, endotracheal intubation status, diabetes mellitus, hemodialysis status, platelet count, international normalized ratio (INR), activated partial thromboplastin time (aPTT), catheter type, and insertion approach. ICU unit was grouped descriptively as surgical ICU, medical ICU, or other adult ICU.

Sex was abstracted from structured clinical records as recorded in the medical record and was analyzed as a biological variable. Gender identity data were not available in the source dataset. Catheter type was abstracted as recorded in the procedure database. Coagulation-related laboratory fields were retained for descriptive baseline characterization but were not included in the primary adjusted model, which was intentionally parsimonious because of the limited number of primary events.

### 2.4. Pleural Effusion Phenotype Grouping

The primary exposure was a prespecified pragmatic pleural effusion phenotype grouping derived from source-database categories. Original recorded categories were transudative effusion, exudative effusion, empyema, malignant pleural effusion, malignancy-related effusion, hemothorax, or unknown. For the primary comparative analysis, exudative effusion, empyema, malignant pleural effusion, and malignancy-related effusion were grouped as complex. Transudative effusion and hemothorax were grouped as simple.

This grouping was intended for procedure-level risk stratification and not as a claim that all component diagnoses share the same biological mechanism. Empyema, malignant pleural effusion, and noninfectious exudative effusions differ in pathophysiology, expected drainage behavior, and downstream management. The grouping instead reflects the practical bedside concern that certain pleural processes may be more likely to have loculation, recurrent fluid production, incomplete drainage, or a need for escalation compared with uncomplicated transudative effusions. Because hemothorax may be considered complex in some clinical frameworks, an exploratory sensitivity analysis reclassified hemothorax as complex to test whether the primary phenotype association was robust to this alternative categorization.

### 2.5. Ultrasound Guidance Approach

Pigtail pleural catheters were placed in the ICU according to local clinical practice. Real-time ultrasound-guided insertion was defined as continuous thoracic ultrasound visualization during pleural access and catheter placement. Ultrasound-assisted insertion was defined as pre-procedure thoracic ultrasound localization of the effusion with selection of the insertion site and estimated depth before skin preparation, followed by catheter placement without continuous real-time ultrasound visualization.

This operational distinction was central to the analysis. Both approaches use ultrasound information, but they differ in how ultrasound is integrated into needle and catheter advancement. The ultrasound-assisted approach may be used in settings where the effusion is localized before the procedure, whereas real-time ultrasound-guided insertion preserves continuous visualization during pleural access. The study did not assign the approach; therefore, the observed association between insertion approach and outcome may reflect selection, case complexity, operator preference, or other unmeasured procedural factors.

### 2.6. Outcome Definitions

The primary outcome was episode-level unplanned catheter removal. It was pragmatically defined as catheter removal for a documented catheter-related complication or escalation to surgery. The composite outcome was selected to capture non-elective deviation from the initial catheter-drainage course during the catheter episode. Because these two components may represent different clinical processes, removal due to complications and escalation to surgery were also reported separately as secondary descriptive outcomes.

This definition was selected before analysis because it aligns with ICU decision-making. A catheter episode that ends because of malposition, pneumothorax, poor position, or another documented complication has deviated from the intended bedside drainage plan. Similarly, a catheter episode that proceeds to surgical escalation represents failure of the initial catheter strategy to remain definitive management for that pleural process. The endpoint does not imply that all events were preventable, nor does it imply that escalation to surgery was inappropriate. Rather, it identifies episodes in which the catheter course was not completed electively.

Planned removal was defined as removal for no ongoing indication. Catheter dwell time was analyzed using the recorded catheter-duration field and is reported in days. Mechanical complications were obtained from the structured complication field and classified as pneumothorax or catheter malposition/poor position when present. Catheter removal reason was obtained from the structured removal-reason field. No separate outcome-adjudication process beyond structured clinical documentation was performed.

### 2.7. Missing Data

Missingness was assessed before analysis. Variables required for cohort assembly and the primary adjusted model—placement date, removal date, removal reason, age, sex, insertion approach, and pleural effusion classification after exclusion of unknown classification—were complete in the analyzed episodes. The primary outcome analysis therefore used a complete-case approach within the prespecified primary analysis set.

Missing values were present in additional pleural fluid laboratory fields, including pleural lactate dehydrogenase, serum lactate dehydrogenase, pleural fluid analysis, and culture results. These variables were not imputed and were not prespecified for the primary adjusted model. When such variables were reported descriptively, variable-specific denominators were used.

### 2.8. Statistical Analysis

All analyses were performed at the catheter-episode level. Categorical variables were summarized as *n*/N (%) and continuous variables as mean (standard deviation) or median [interquartile range], as appropriate. Between-group comparisons used the chi-square test or Fisher exact test for categorical variables and the Student t test or Wilcoxon rank-sum test for continuous variables, as appropriate.

For the primary endpoint, an adjusted analysis compared complex versus simple pleural effusion groupings. Because unplanned catheter removal was infrequent, Firth penalized logistic regression was used for the primary model. The binary outcome was unplanned catheter removal, and prespecified predictors were effusion phenotype grouping, age per one-year increase, sex, and insertion approach (ultrasound-assisted versus real-time ultrasound-guided). No data-driven variable selection was performed. Effect estimates from the adjusted model are reported as adjusted odds ratios (aORs) with 95% confidence intervals (CIs). All *p* values were two-sided, and *p* < 0.05 was considered statistically significant.

Because of the limited number of outcome events, the adjusted model was intentionally parsimonious and should be interpreted as estimating adjusted associations rather than as a fully confounder-adjusted causal model [17,18]. Secondary outcomes were summarized descriptively; no separate adjusted models were prespecified for these endpoints. A prespecified sensitivity analysis repeated the same model after restricting the dataset to the first eligible catheter placement episode per patient. Because of the low event count, no clustering-adjusted model was prespecified for the primary analysis [19]. All analyses were performed in Python 3.11.2 using pandas 2.2.3, NumPy 1.24.0, and SciPy 1.14.1. Firth penalized logistic regression was implemented by solving the modified score equations with Jeffreys-prior penalization, and standard errors were derived from the penalized Fisher information matrix.

No post hoc expansion of the adjustment set was performed. This decision was conservative: adding many covariates to a sparse-event model can create unstable estimates that appear precise only because the model is overfit. Clinically relevant variables such as intubation status, hemodialysis, coagulation parameters, catheter type, and ICU unit were therefore presented descriptively rather than forced into the primary model. The model should be read as a structured signal-detection analysis focused on the prespecified exposure and ultrasound workflow, not as a comprehensive causal adjustment strategy.

### 2.9. Ethics

This study was approved by the MacKay Memorial Hospital Institutional Review Board (IRB No. 25MMHIS036e; approval date: 1 April 2025). The requirement for informed consent was waived because of the retrospective design and use of existing de-identified clinical data. The study was conducted in accordance with the Declaration of Helsinki.

## 3. Results

### 3.1. Study Flow and Analysis Population

**Figure 1:**
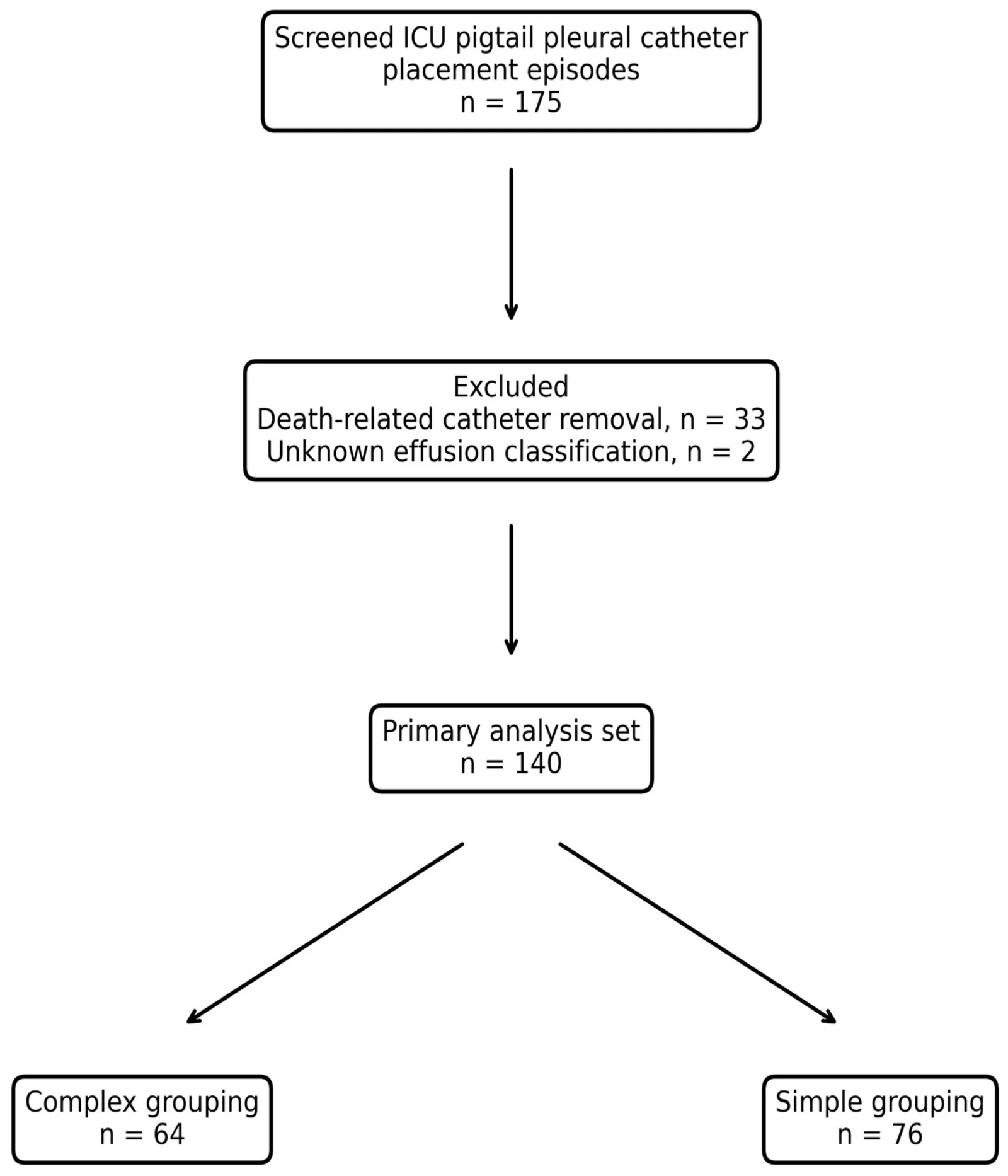
Cohort flow diagram. The flow diagram summarizes cohort derivation from 175 screened pigtail pleural catheter placement episodes to the primary analysis set (*n* = 140) after excluding episodes with catheter removal recorded as death (*n* = 33) and episodes with unknown pleural effusion classification (*n* = 2).

During the study period, 175 pigtail pleural catheter placement episodes were screened. Thirty-three episodes were excluded because catheter removal was recorded as death, and two episodes were excluded because pleural effusion classification was unknown. The primary analysis set therefore included 140 catheter placement episodes. Of these, 64/140 episodes (45.7%) were classified as complex and 76/140 episodes (54.3%) were classified as simple (Figure 1).

The prespecified first-episode-per-patient sensitivity cohort contained 118 episodes, corresponding to 118 unique patients. Thus, the full primary cohort included 22 repeat procedures beyond the first eligible episode. The source dataset did not reliably distinguish whether all repeat procedures occurred within the same hospitalization or across separate hospitalizations.

### 3.2. Baseline Characteristics

Baseline demographic, clinical, and procedural characteristics are summarized in Table 1. In the primary analysis set, mean age was 69.7 years with a standard deviation of 15.3 years, and 77/140 episodes (55.0%) occurred in male patients. Endotracheal intubation was present in 85/140 episodes (60.7%). Diabetes mellitus and hemodialysis were recorded in 45/140 episodes (32.1%) and 12/140 episodes (8.6%), respectively.

Real-time ultrasound-guided and ultrasound-assisted insertion approaches were used in 71/140 episodes (50.7%) and 69/140 episodes (49.3%), respectively. Catheter type was COOK in 103/140 episodes (73.6%) and wire-locked in 37/140 episodes (26.4%). Baseline characteristics were broadly similar between complex and simple groups, although the study was not powered to detect small imbalances in baseline features.

### 3.3. Primary Outcome

Unplanned catheter removal occurred in 12/140 episodes (8.6%) overall and was more frequent in the complex group than in the simple group (10/64 [15.6%] vs. 2/76 [2.6%]) (Table 2). The composite primary outcome included 7/140 episodes (5.0%) with removal due to catheter-related complications and 5/140 episodes (3.6%) with escalation to surgery. All surgical escalations occurred in the complex group.

**Figure 2:**
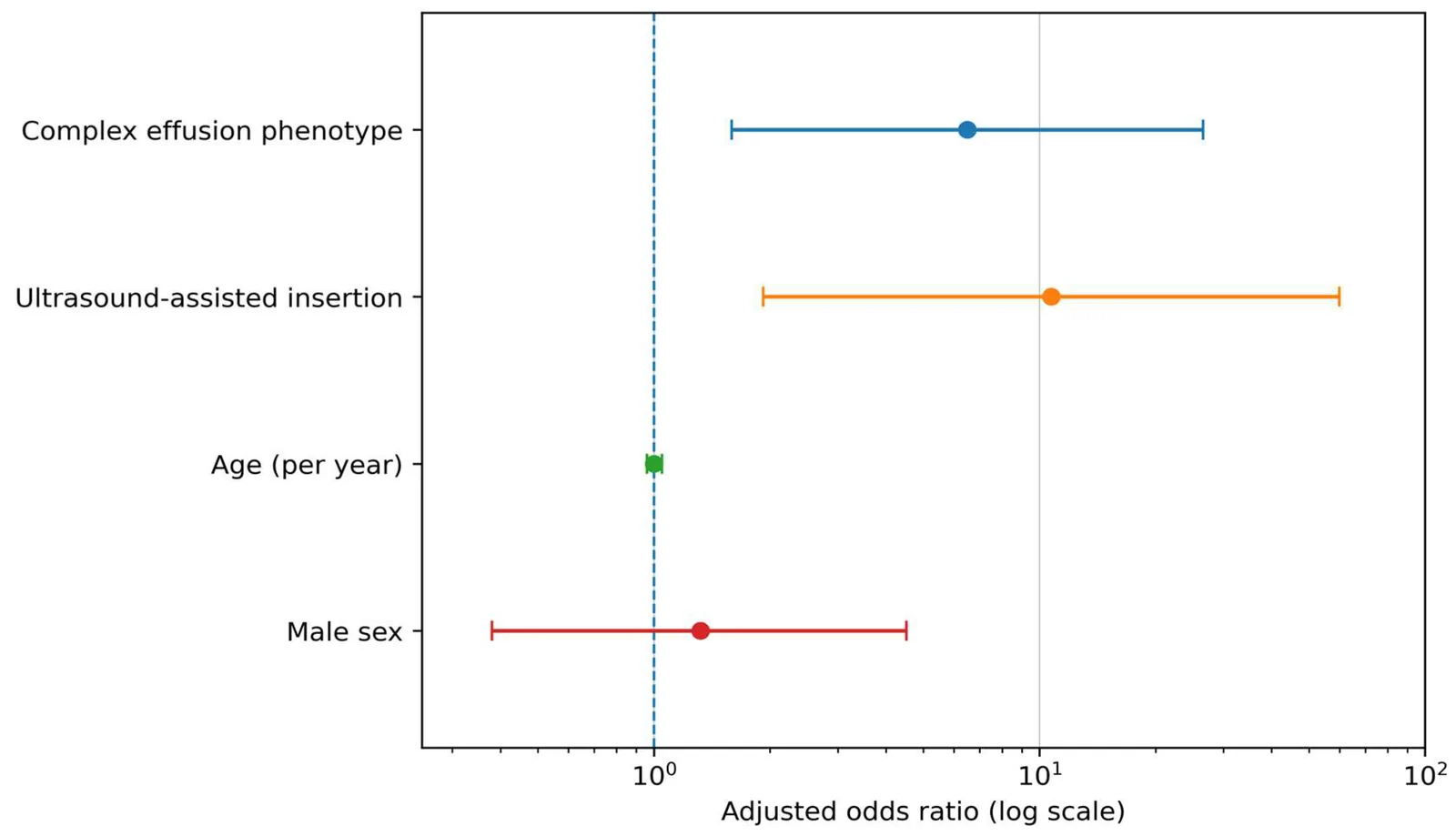
Adjusted odds ratios (aORs) for unplanned catheter removal in the primary analysis set. The forest plot shows aORs with 95% confidence intervals for complex effusion grouping, ultrasound-assisted insertion (vs. real-time ultrasound-guided), age (per year), and male sex. The *x*-axis is displayed on a logarithmic scale (The blue dashed vertical line indicates the null value (aOR = 1)).

In adjusted analyses, complex effusion grouping was associated with higher odds of unplanned catheter removal (aOR 6.50, 95% CI 1.59–26.55; *p* = 0.009). Ultrasound-assisted insertion was also associated with higher odds of unplanned catheter removal compared with real-time ultrasound-guided insertion (aOR 10.72, 95% CI 1.92–59.94; *p* = 0.007). Age (aOR 1.00, 95% CI 0.96–1.05; *p* = 0.843) and male sex (aOR 1.32, 95% CI 0.38–4.52; *p* = 0.663) were not associated with the primary outcome in the adjusted model (Table 3 and Figure 2).

### 3.4. Secondary Outcomes and Sensitivity Analyses

**Figure 3:**
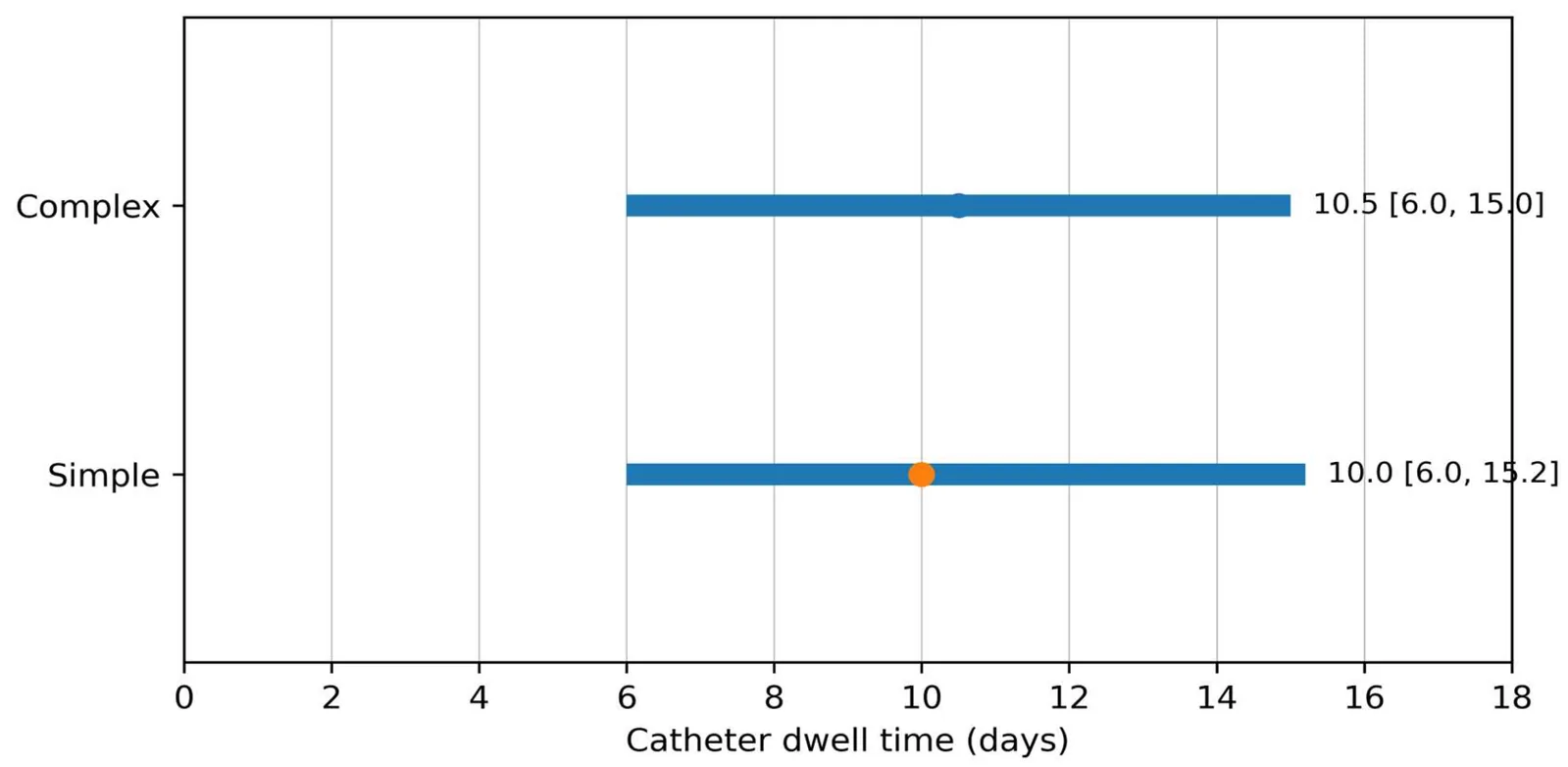
Catheter dwell time by effusion grouping in the primary analysis set. The interval plot shows the median and interquartile range of catheter dwell time in days for the complex and simple groups.

Any recorded mechanical complication occurred in 8/140 episodes (5.7%) overall, including pneumothorax in 3/140 episodes (2.1%) and catheter malposition/poor position in 5/140 episodes (3.6%). Median catheter dwell time was 10.0 [6.0, 15.0] days overall, 10.5 [6.0, 15.0] days in the complex group, and 10.0 [6.0, 15.2] days in the simple group (Table 2 and Figure 3).

In descriptive analyses by original phenotype subtype, unplanned removal occurred in 6/34 exudative effusions (17.6%), 2/15 empyemas (13.3%), 2/12 malignant pleural effusions (16.7%), 0/3 malignancy-related effusions, 2/64 transudative effusions (3.1%), and 0/12 hemothoraces (Supplementary Table S1). In the exploratory sensitivity analysis reclassifying hemothorax as complex, the association between complex grouping and unplanned removal remained directionally similar but was attenuated (aOR 4.84, 95% CI 1.17–19.93; *p* = 0.029), whereas the association for ultrasound-assisted insertion was materially unchanged (aOR 10.49, 95% CI 1.89–58.34; *p* = 0.007) (Supplementary Table S2).

In the prespecified sensitivity analysis restricted to the first eligible episode per patient (n = 118), unplanned catheter removal occurred in 10/118 episodes (8.5%) overall and remained more frequent in complex than simple episodes (8/56 [14.3%] vs. 2/62 [3.2%]). Adjusted associations for complex effusion grouping and ultrasound-assisted insertion were directionally consistent with the primary analysis (Table 3).

## 4. Discussion

### 4.1. Principal Findings

In this single-center adult ICU episode-level cohort, a pragmatic complex effusion phenotype and ultrasound-assisted, rather than real-time ultrasound-guided, insertion approach were associated with higher odds of unplanned pigtail catheter removal. The association persisted directionally in the sensitivity analysis restricted to the first eligible episode per patient. In the exploratory analysis that reclassified hemothorax as complex, the phenotype estimate was attenuated (aOR 4.84, 95% CI 1.17–19.93) but remained directionally consistent, underscoring both robustness and the limitations of treating hemothorax as uniformly simple. These results should be interpreted as signals of catheter-course vulnerability rather than as definitive evidence of technical failure or causal harm from any specific approach. The primary outcome was deliberately pragmatic: it captured removal for catheter-related complications and escalation to surgery, two clinically important ways in which the initial catheter-drainage pathway may fail to proceed as planned.

### 4.2. Interpretation in the Context of Pleural Disease and Ultrasound Literature

The association between complex effusion grouping and unplanned removal is clinically plausible. Pleural infection can evolve from free-flowing fluid to septated, loculated, and organized collections, and randomized evidence has shown that adjunctive intrapleural therapy may be required in selected cases [5,6,20,21]. Malignant pleural effusion poses a different challenge, in which recurrence, symptom burden, pleural apposition, trapped lung physiology, and patient goals influence whether repeated procedures, indwelling pleural catheter strategies, pleurodesis, or other interventions are favored [7,22,23]. The present study did not attempt to model these pathways separately because event counts were low, but the descriptive phenotype-level results support the broader hypothesis that pleural etiology influences the downstream catheter course.

The ultrasound finding deserves especially careful interpretation. The observed association between ultrasound-assisted insertion and unplanned catheter removal should not be read as a conclusion that ultrasound is harmful. Ultrasound is foundational to safe pleural procedures, and multiple guidelines and statements emphasize its role in planning, localization, and procedural safety [9,10,11,12,24]. The more likely interpretation is that ultrasound workflow may be a proxy for procedural complexity, selection practice, operator workflow, or local bedside constraints. Pre-procedure localization may be selected in cases that appear straightforward, but it may also be used in operational circumstances where continuous real-time visualization is not maintained. Conversely, real-time guidance may provide continuous feedback during pleural access, which could be advantageous when the fluid pocket is small, shifting, loculated, or close to adjacent structures. The current dataset cannot separate these mechanisms. Residual confounding by indication, operator practice, and procedural workflow therefore cannot be excluded; this association should not be used to rank ultrasound approaches or to infer that one approach causes catheter-course failure.

Several alternative explanations are plausible. Operators may have chosen ultrasound-assisted insertion when the effusion had already been localized by a prior scan, when the bedside setup made continuous visualization difficult, or when clinical urgency favored a faster workflow. Conversely, operators may have used real-time guidance for cases in which they anticipated difficulty and therefore maintained continuous imaging to reduce uncertainty. Without granular data on effusion depth, patient positioning, respiratory variation, operator experience, procedural urgency, and ultrasound image quality, the statistical association cannot identify which explanation is correct. This is precisely why the result should change documentation behavior before it changes procedural doctrine.

### 4.3. Endpoint Interpretation and Clinical Implications

Most of the ultrasound literature in pleural procedures has focused on thoracentesis and immediate complications such as pneumothorax [25,26,27,28]. Our study differs because it examines pigtail catheter episodes rather than a single aspiration procedure. A catheter episode includes access, placement, securing, drainage performance, clinical reassessment, and eventual removal. Immediate procedural success can therefore coexist with later catheter-course failure. Earlier reports demonstrated that ultrasound-guided pigtail drainage is feasible across pleural disease categories, but they did not primarily evaluate unplanned catheter removal as an episode-level outcome [29,30]. More recent evidence indicates that complication and repeat-intervention rates vary by pleural effusion etiology, supporting the concept that patient and effusion phenotype may modify catheter outcomes [31,32,33].

The composite endpoint is both a strength and a limitation. It is a strength because ICU clinicians care about deviations from the intended drainage plan, not only narrowly defined mechanical complications. A patient who requires surgical escalation after pigtail placement represents a clinically important catheter-course event even if the initial catheter was technically positioned. It is a limitation because complication-driven removal and surgical escalation may have different mechanisms, clinical severity, and implications. Composite endpoints can amplify interpretive ambiguity when component frequencies differ or when one component dominates the association [34]. In this cohort, all surgical escalations occurred in complex effusions, whereas complication-related removals occurred in both groups. Future studies should have sufficient sample size to model individual components separately.

The small number of events is the central statistical constraint. Only 12 unplanned catheter removals occurred in the primary analysis set. Firth penalized logistic regression was selected to reduce small-sample bias in sparse binary outcome modeling, and the adjusted model was intentionally parsimonious [17,18]. Even so, the wide confidence intervals indicate limited precision. The results therefore should be used for hypothesis generation, quality-improvement prioritization, and study design rather than for definitive bedside prediction or a change in clinical practice. From an operational perspective, the effect sizes are important enough to justify better prospective data capture, but not stable enough to support hard policy mandates. The signal is a flare on the dashboard, not the final root-cause analysis.

### 4.4. Strengths, Limitations, and Future Directions

A clinically reasonable response is therefore tiered rather than absolutist. Complex effusion phenotype or ultrasound-assisted placement should not automatically trigger surgical referral, larger-bore tube placement, or a change in device choice. Instead, these factors can identify episodes that merit clearer plans: when to reassess drainage, when to repeat ultrasound or chest imaging, what output threshold would be considered inadequate, and which clinical deterioration signs should prompt escalation. In other words, the study supports a risk-aware surveillance pathway, not a reflexive intervention pathway.

Several clinical implications follow from a cautious reading of the findings. First, documentation should distinguish ultrasound-assisted localization from real-time ultrasound-guided insertion rather than collapsing both into a generic ultrasound category. Second, patients with complex effusion phenotypes may benefit from early post-placement reassessment, including reassessment of drainage output, symptoms, ventilator status, catheter position, and repeat imaging when clinically indicated. Third, teams should define escalation triggers early in the catheter course. For example, persistent sepsis, poor drainage despite radiographic residual collection, suspected loculation, catheter malposition, or recurrent fluid accumulation may prompt review of catheter position, intrapleural therapy, additional drainage, or surgical consultation depending on etiology and patient goals. These implications remain inferential and should be validated prospectively.

The study has several strengths. It focuses on adult ICU patients, in whom pleural drainage decisions are embedded in acute respiratory and hemodynamic management. It uses a procedure-level analytic framework, which aligns with how catheter exposures and outcomes were recorded. It distinguishes ultrasound-assisted from real-time ultrasound-guided insertion, an operational detail that is often missing from retrospective datasets. It also includes a first-episode-per-patient sensitivity analysis to evaluate whether repeated episodes were driving the association.

The limitations are substantial and should be explicit. First, the retrospective single-center design introduces confounding by indication and limits generalizability. Second, operator experience, procedural urgency, patient positioning, effusion size, ultrasound appearance, catheter securing method, anticoagulant exposure, and post-placement management were not fully captured in the adjusted model. Third, the complex effusion phenotype grouped biologically distinct entities; exudative effusion, empyema, malignant pleural effusion, and malignancy-related effusion should not be assumed to have the same mechanism of catheter-course failure. Fourth, outcome ascertainment relied on structured documentation of complications and removal reason, which may miss mixed or evolving reasons for catheter discontinuation. Fifth, the episode-level framework allowed for repeated procedures from some patients, and although the first-episode sensitivity analysis was directionally consistent, unmodeled within-patient clustering may still have affected precision [19]. Finally, exclusion of death-related catheter removals defined a classifiable catheter-course cohort but did not formally address death as a competing event [35].

The death-related exclusion deserves particular attention in an ICU study. Patients who die with a pleural catheter in place may not live long enough to experience catheter removal for complication, failure, or surgical escalation. Excluding such episodes avoids misclassifying death as planned catheter completion, but it also narrows inference to episodes in which a catheter-course endpoint could be observed and classified. Accordingly, the observed 8.6% unplanned removal rate is conditional on the 140 episodes with a classifiable non-death catheter-course endpoint and should not be interpreted as the cumulative incidence among all 175 screened episodes. Because death precluded observation of later catheter-course events in 33 episodes, the direction and magnitude of any resulting selection bias cannot be determined from these data. A competing-risk framework would be more appropriate in larger prospective datasets, especially if the goal is to estimate absolute risks over time rather than adjusted associations among classifiable episodes.

The handling of hemothorax illustrates why pragmatic phenotype groupings require clinical humility. Hemothorax can be simple in the sense that it may represent a mechanically drainable pleural collection, but it can also become complex when clot organization, retained hemothorax, or ongoing bleeding changes the management pathway. In the primary grouping, hemothorax was categorized with simple effusions because no unplanned removals occurred among the recorded hemothorax episodes and because the grouping was defined for the main prespecified analysis. The exploratory reclassification showed attenuation of the complex effusion phenotype association but did not reverse it. This supports robustness, but it also warns against treating any binary classification as biologically absolute.

The findings are also relevant to institutional quality improvement. A low event rate can still represent meaningful operational risk when the event triggers repeat procedures, surgical consultation, or prolonged drainage. A practical implementation step would be to create a standardized pleural catheter note template that records effusion phenotype, sonographic complexity, whether ultrasound use was assisted or real-time-guided, catheter type, securing method, immediate complications, planned reassessment timing, and explicit escalation criteria. Such a template would improve clinical communication today and create cleaner data for tomorrow. That is the type of small systems investment that prevents retrospective databases from becoming archaeological digs with stethoscopes.

External validity requires caution. The study reflects one hospital, one procedural culture, and one documentation ecosystem. ICUs with interventional pulmonology support, radiology-led drainage pathways, different catheter sizes, different securing protocols, or routine intrapleural therapy may observe different event rates and different effect estimates. Nevertheless, the conceptual framework should travel well: pleural phenotype and ultrasound workflow are measurable variables, and both are plausible determinants of catheter-course outcomes. The immediate value of this study is therefore not a universal prediction model, but a reproducible classification strategy that other centers can test.

Future prospective studies should capture a richer procedural and pleural phenotype dataset. Useful fields would include sonographic complexity, loculation, septations, estimated effusion depth, insertion site, patient position, real-time visualization during each step, operator training level, catheter French size and securing method, anticoagulation status, drainage-output trajectory, repeat imaging, intrapleural therapy, and reason-specific removal adjudication. Multicenter datasets would also allow for more stable modeling of individual event components and clinically meaningful interactions between etiology, ultrasound workflow, and catheter strategy. Given the broader epidemiologic burden of pleural disease, improved procedural datasets could help move pleural drainage research from descriptive safety reporting toward actionable risk stratification [36,37].

## 5. Conclusions

In this single-center adult ICU cohort, a pragmatic complex effusion phenotype and ultrasound-assisted pigtail insertion approach were associated with a higher likelihood of unplanned catheter removal. These findings are hypothesis-generating and are not sufficient to support changes in clinical practice because of the sparse event count, wide confidence intervals, retrospective design, potential confounding by indication, and composite endpoint. Prospective multicenter studies with standardized ultrasound workflow documentation and adjudicated catheter-course outcomes are needed to validate these associations and determine whether targeted reassessment or escalation pathways can improve pleural drainage outcomes in critically ill adults.

## Figures and Tables

**Table 1:** Baseline characteristics of the primary analysis set (n = 140) by effusion grouping.

Characteristic	Overall (*n* = 140)	Complex (*n* = 64)	Simple (*n* = 76)
Age, years	69.7 (15.3)	70.4 (13.0)	69.1 (17.1)
Male sex	77/140 (55.0%)	37/64 (57.8%)	40/76 (52.6%)
Endotracheal intubation	85/140 (60.7%)	41/64 (64.1%)	44/76 (57.9%)
ICU type: surgical ICU	71/140 (50.7%)	34/64 (53.1%)	37/76 (48.7%)
ICU type: medical ICU	17/140 (12.1%)	6/64 (9.4%)	11/76 (14.5%)
ICU type: other adult ICU	52/140 (37.1%)	24/64 (37.5%)	28/76 (36.8%)
Right-sided effusion	78/140 (55.7%)	38/64 (59.4%)	40/76 (52.6%)
Diabetes mellitus	45/140 (32.1%)	21/64 (32.8%)	24/76 (31.6%)
Hemodialysis	12/140 (8.6%)	3/64 (4.7%)	9/76 (11.8%)
Platelet count, ×10^9^/L	191 [103, 316]	210 [103, 342]	189 [105, 293]
INR	1.16 [1.08, 1.25]	1.16 [1.04, 1.25]	1.16 [1.09, 1.27]
aPTT, s	30.2 [27.6, 36.9]	31.1 [27.8, 38.0]	30.0 [27.6, 36.2]
Insertion approach: real-time ultrasound-guided	71/140 (50.7%)	34/64 (53.1%)	37/76 (48.7%)
Insertion approach: ultrasound-assisted	69/140 (49.3%)	30/64 (46.9%)	39/76 (51.3%)
Catheter type: COOK	103/140 (73.6%)	47/64 (73.4%)	56/76 (73.7%)
Catheter type: wire-locked	37/140 (26.4%)	17/64 (26.6%)	20/76 (26.3%)

Values are presented as mean (SD), median [IQR], or n/N (%). Complex grouping includes exudative effusion, empyema, malignant pleural effusion, and malignancy-related effusion. Simple grouping includes transudative effusion and hemothorax. aPTT, activated partial thromboplastin time; ICU, intensive care unit; INR, international normalized ratio; IQR, interquartile range; SD, standard deviation.

**Table 2:** Primary and secondary outcomes in the primary analysis set (*n* = 140) by effusion grouping.

Outcome	Overall (*n* = 140)	Complex (*n* = 64)	Simple (*n* = 76)
Unplanned catheter removal	12/140 (8.6%)	10/64 (15.6%)	2/76 (2.6%)
Removal due to complications	7/140 (5.0%)	5/64 (7.8%)	2/76 (2.6%)
Escalation to surgery	5/140 (3.6%)	5/64 (7.8%)	0/76 (0.0%)
Planned removal (no indication)	128/140 (91.4%)	54/64 (84.4%)	74/76 (97.4%)
Any recorded mechanical complication	8/140 (5.7%)	6/64 (9.4%)	2/76 (2.6%)
Pneumothorax	3/140 (2.1%)	2/64 (3.1%)	1/76 (1.3%)
Catheter malposition/poor position	5/140 (3.6%)	4/64 (6.2%)	1/76 (1.3%)
Catheter dwell time, days	10.0 [6.0, 15.0]	10.5 [6.0, 15.0]	10.0 [6.0, 15.2]

Values are presented as n/N (%) or median [IQR]. Complex grouping includes exudative effusion, empyema, malignant pleural effusion, and malignancy-related effusion. Simple grouping includes transudative effusion and hemothorax. IQR, interquartile range.

**Table 3:** Adjusted associations with unplanned catheter removal.

Predictor	Primary Analysis Set (*n* = 140)	First Episode per Patient (*n* = 118)
Complex effusion grouping	6.50 (1.59–26.55); *p* = 0.009	4.54 (1.07–19.20); *p* = 0.040
Ultrasound-assisted insertion	10.72 (1.92–59.94); *p* = 0.007	8.35 (1.48–47.25); *p* = 0.016
Age (per year)	1.00 (0.96–1.05); *p* = 0.843	1.01 (0.96–1.06); *p* = 0.682
Male sex	1.32 (0.38–4.52); *p* = 0.663	1.43 (0.38–5.34); *p* = 0.596

Values are adjusted odds ratios (aORs) with 95% confidence intervals and two-sided *p* values. The primary analysis set model uses all eligible episodes, and the sensitivity model is restricted to the first eligible episode per patient. Given the small number of primary events, estimates should be interpreted cautiously.

## Data Availability

The data presented in this study are available on request from the corresponding author due to privacy and ethical restrictions. The data are not publicly available because they contain institutional clinical records that cannot be openly shared under the approved ethical framework.

## Supplementary Materials

The following supporting information can be downloaded at: https://www.mdpi.com/article/10.3390/life16081331/s1, Table S1. Outcome frequencies stratified by original phenotype subtype in the primary analysis set (*n* = 140); Table S2. Exploratory sensitivity analysis reclassifying hemothorax as complex in the primary adjusted model (*n* = 140).

## Abbreviations

aOR, adjusted odds ratio; aPTT, activated partial thromboplastin time; CI, confidence interval; EHR, electronic health record; ICU, intensive care unit; INR, international normalized ratio; IQR, interquartile range; SD, standard deviation; STROBE, Strengthening the Reporting of Observational Studies in Epidemiology.
