# Supplementary material for: Pleural Effusion Phenotype and Ultrasound Guidance Approach Associated with Unplanned Pigtail Pleural Catheter Removal in an Adult Intensive Care Unit: A Single-Center Retrospective Cohort Study
Source: Life (Basel). 2026 Aug 14;16(8):1331. doi: 10.3390/life16081331 (PMC13514574; doi:10.3390/life16081331)
Supplement: Supplementary file 1 [file life-16-01331-s001.zip › life-4458555-supplementary.pdf]

Supplementary Tables

Pleural Effusion Phenotype and Ultrasound Guidance Approach Associated With Unplanned Pigtail Pleural Catheter Removal in an Adult Intensive Care Unit: A Single-Center Retrospective Cohort Study

Wei-Hung Chang[1,2], Kuan-Pen Yu[1], Li-Kuo Kuo[1], Chung Lee[3,*]

Table S1. Outcome frequencies stratified by original phenotype subtype in the primary analysis set (n = 140).

Original phenotype subtype	Episodes, n	Unplanned catheter removal	Removal due to complications	Escalation to surgery	Planned removal (no indication)	Any recorded mechanical complication
Exudative effusion	34	6/34 (17.6%)	4/34 (11.8%)	2/34 (5.9%)	28/34 (82.4%)	5/34 (14.7%)
Empyema	15	2/15 (13.3%)	0/15 (0.0%)	2/15 (13.3%)	13/15 (86.7%)	0/15 (0.0%)
Malignant pleural effusion	12	2/12 (16.7%)	1/12 (8.3%)	1/12 (8.3%)	10/12 (83.3%)	1/12 (8.3%)
Malignancy-related effusion	3	0/3 (0.0%)	0/3 (0.0%)	0/3 (0.0%)	3/3 (100.0%)	0/3 (0.0%)
Transudative effusion	64	2/64 (3.1%)	2/64 (3.1%)	0/64 (0.0%)	62/64 (96.9%)	2/64 (3.1%)
Hemothorax	12	0/12 (0.0%)	0/12 (0.0%)	0/12 (0.0%)	12/12 (100.0%)	0/12 (0.0%)

The primary analysis set excluded episodes removed because of death and episodes with unknown effusion phenotype.

Table S2. Exploratory sensitivity analysis reclassifying hemothorax as complex in the primary adjusted model (n = 140).

Predictor	Original primary model, OR (95% CI); p value	Hemothorax reclassified as complex, OR (95% CI); p value
Complex grouping	6.50 (1.59-26.55); p = 0.009	4.84 (1.17-19.93); p = 0.029
Age (per year)	1.00 (0.96-1.05); p = 0.843	1.01 (0.96-1.05); p = 0.708
Male sex	1.32 (0.38-4.52); p = 0.663	1.50 (0.45-5.05); p = 0.512
Ultrasound-assisted insertion	10.72 (1.92-59.94); p = 0.007	10.49 (1.89-58.34); p = 0.007

Both models used Firth penalized logistic regression adjusted for age, sex, and insertion approach. The reclassification analysis was exploratory and intended to test the robustness of the phenotype association to alternative handling of hemothorax.
